# Professional and public views about early return of patients from Comprehensive Stroke Centers to local Acute Stroke Centers in England following displacement by emergency care pathways

**DOI:** 10.3389/fstro.2024.1431799

**Published:** 2024-11-20

**Authors:** Chris Price, Becky Zhu, Abigail Alton, Gary A. Ford, Martin James, Graham McClelland, Phil White, Lisa Shaw

**Affiliations:** ^1^Stroke Research Group, Newcastle University, Newcastle upon Tyne, United Kingdom; ^2^University of Oxford, Oxford, United Kingdom; ^3^Royal Devon and Exeter Hospital, Exeter, United Kingdom; ^4^University of Exeter Medical School and National Institute for Health and Care Research (NIHR) South West Peninsula Applied Research Collaboration, Exeter, United Kingdom; ^5^Faculty of Health and Life Sciences, Northumbria University, Newcastle upon Tyne, United Kingdom

**Keywords:** stroke, stroke care access, thrombectomy [MeSH], care pathway, service organization, patient safety

## Abstract

**Background:**

Mechanical thrombectomy is a highly effective emergency treatment for selected cases of ischemic stroke but can only be provided at hospitals with appropriate facilities and interventionists. Many patients require transfers for treatment, including some who are subsequently considered ineligible. To maintain capacity at thrombectomy centers, displaced patients should soon be returned to their local hospital following assessment and treatment, but return processes vary. We sought the views of stroke and ambulance services, clinicians, and public representatives about the timing, planning and implementation of acceptable processes to inform recommendations about the early return of patients (< 24 h) displaced as a result of thrombectomy pathways.

**Methods:**

Three workstreams were undertaken between 01/05/2023 and 31/10/2023: 1. An online survey of hospital stroke services supplemented by a convenience poll of stroke clinicians. 2. An online survey of ambulance services. 3. Focus groups with stroke patients and carers using a topic guide describing typical early return scenarios. The surveys used multiple choice answers supplemented by free text boxes for additional comments. Data were reported descriptively without statistical comparison. Focus group data were analyzed thematically using emergent coding.

**Results:**

Responses were obtained from 32 stroke services, 44 stroke clinicians, and 11 ambulance services. Stroke service and clinician respondents generally supported early return for most clinical scenarios but advised caution regarding transfers < 4 h after thrombectomy and < 24 h for hemorrhagic stroke due to the higher risk of complications. Ambulance respondents highlighted travel time, immediate service pressures and crew type as influences upon providing early returns, but supported 24/7 provision. Twenty-nine patients and four carers participated in three focus groups. There was general acceptance of early return processes but these participants emphasized the need for clear communication and individualized decisions based upon clinical status, age, journey length, patient preferences and individual contextual factors.

**Conclusions:**

All contributors were generally supportive of early patient returns to maintain thrombectomy center capacity, but the results suggest important organizational, clinical, and patient-focused considerations for successful implementation.

## 1 Background

Stroke is a common cause of severe adult disability (Feigin et al., 2014; Xu et al., 2018) and death (Stroke Association, 2018), but outcomes can be significantly improved by specialist care including emergency treatments for selected patients (Stroke Unit Trialists Collaboration, 2013; Wardlaw et al., 2014; Saver et al., 2016; Goyal et al., 2016). The most powerful of these is mechanical thrombectomy for disabling ischemic stroke, an emergency procedure performed by neuro-interventionists to remove blood clots (thrombus) blocking large blood vessels supplying the brain (Saver et al., 2016; Goyal et al., 2016). Up to 15% of stroke patients are suitable for thrombectomy (McMeekin et al., 2024), but in many healthcare systems this treatment is only available in a limited number of regional Comprehensive Stroke Centers (CSC) with the necessary facilities, specialist workforce and volume of activity to maintain interventionist skills (Ciccone et al., 2019; Sentinel Stroke National Audit Programme, 2024).

Stroke patients living near to a CSC have direct access to thrombectomy, but as CSCs do not usually have the capacity to admit all cases of suspected stroke across a region, many patients needing thrombectomy are transferred from primary Acute Stroke Centers (ASCs) following initial clinical and radiological assessment. For instance, in England there are 24 established CSC whereas ~70% of stroke patients are first admitted to a nearer ASC and rapid transfer to a CSC is necessary if thrombectomy is appropriate (Sentinel Stroke National Audit Programme, 2024; Allen et al., 2019). This is referred to as “drip and ship” because such patients are often also suitable for intravenous thrombolysis drug treatment, which is commenced at the ASC prior to transfer. An additional admission route to CSC in some settings is through prehospital screening assessments to identify suspected stroke patients with characteristics suggesting possible suitability for thrombectomy, which trigger ambulance bypass of the local ASC in favor of the CSC (Mazya et al., 2020; Brandler et al., 2024). However, clinical review and investigations at the CSC show that many of these patients are unsuitable for thrombectomy, and the care required could have been provided at the local hospital.

A consequence of any emergency pathway to access centralized therapies is that patients are displaced from their local care setting, and often require transfer back for ongoing care and rehabilitation. For a stroke thrombectomy pathway, this can comprise a heterogeneous group of individuals including those who received thrombectomy with and without clinical improvement, those who did not receive thrombectomy because the type of stroke was not suitable for treatment (e.g., hemorrhagic stroke) or the procedure was not technically possible (e.g., vascular anatomy reasons), patients whose condition improved during transfer, and bypass patients who were found to have a different non-stroke “mimic” condition responsible for their symptoms following additional investigations. For as long as these patients remain at a CSC, the capacity to provide thrombectomy might be reduced, especially on days when there is a surge in demand, and result in an overall negative impact upon efficient use of healthcare resources. Recent qualitative exploration of the views of stroke care professionals in England has strongly highlighted concerns that accumulation of patients at CSCs impedes thrombectomy access (Day et al., 2024), and is likely to be a factor in the current substandard delivery of thrombectomy in the UK [3.9% of all stroke admissions in 2023/24 (Sentinel Stroke National Audit Programme, 2024)]. Displacement from a local hospital for >24 h may also be inconvenient and distressing for patients and families in some settings, as national audit data reports that the median travel time between ASC and CSC is 50 min.

Although provision of thrombectomy in England is recommended by National Clinical Guidelines (Intercollegiate Stroke Working Party, 2023) and healthcare policy (NHS England, 2018), there are currently no recommendations regarding the care of patients who are displaced as a consequence of thrombectomy pathways. There may be positive and negative views from both professionals and the public about early (i.e., < 24 h) patient return processes, also known as rapid repatriation. For some scenarios, clinicians might be concerned about a very early transfer (e.g., < 4 h) because of the development of complications from thrombectomy, thrombolysis or the stroke itself that could occur after patients are moved to a distant and less resourced ASC. Views from ambulance services are equally important, especially if new clinical guidelines or training would be required to ensure patient safety, whilst use of their resources to provide return journeys might cause a temporary reduction in their overall ability to respond to emergency calls. It is also unknown what concerns patients and carers would have about early return processes intended to improve service efficiency, and how this aspect of care should be delivered to minimize discomfort.

## 2 Aim

The aim of this study was to describe the views of English stroke services (CSC and ASC), ambulance services and public representatives (patients and carers) about possible processes for the early return (< 24 h) of patients displaced at CSC as a result of thrombectomy pathways, and to describe clinical characteristics and service factors considered important for implementation by these different stakeholders.

## 3 Methods

Due to the number of stroke and ambulance services nationally, an online survey was considered the most efficient approach for collecting views about possible early return processes from CSCs. However, to ascertain patient and carer views about this complex topic, qualitative exploration with relevant representatives was believed to be a more appropriate method to understand their perspectives. Three workstreams seeking information about relevant aspects of early return pathways according to the type of participants were generated:

1. An online survey of hospital stroke services supplemented by a convenience poll of stroke clinicians.
2. An online survey of ambulance services.
3. Focus groups with patients and carers identified through community groups with an interest in stroke care.

Quantitative survey and poll data were analyzed descriptively without statistical comparison. Focus group data were analyzed thematically using emergent coding. Due to the time available to undertake the project, there was no formal pilot phase for the workstreams.

### 3.1 Hospital stroke services

The hospital survey questions focussed upon (i) operational aspects of patient return processes that would be relevant from a hospital perspective, and (ii) return timescales for different common clinical scenarios. Multiple choice answers supplemented by free text boxes for additional comments were used to collect responses. The questions were developed by a stroke physician (CP) with review by two additional stroke physicians (GAF and MJ) and one interventional neuroradiologist (PW). An online data platform (www.castoredc.com) was used for administration.

For the questions concerning specific return timescales, seven clinical scenarios were generated by examination of national audit data (Sentinel Stroke National Audit Programme, 2024) and published descriptions of CSC populations (Mazya et al., 2020; Brandler et al., 2024; Griffin et al., 2020). These included: ischemic stroke after thrombectomy and/or thrombolysis with and without early improvement, hemorrhagic stroke requiring intravenous blood pressure lowering treatment, any stroke patient not requiring emergency medical treatment, and “mimic” presentations caused by a non-stroke condition. For each scenario, respondents were asked to indicate whether the timescale for return of a patient was acceptable within 4, 4–12, and 12–24 h, or unacceptable < 24 h. Respondents were instructed to assume that patients were medically stable for the duration of a transfer back to their local hospital, and had received all other initial treatment as appropriate. For scenarios indicating a clinical improvement, this was described as at least four points difference on the National Institutes of Health Stroke Score (National Institutes of Health and National Institute of Neurological Disorders and Stroke, 2024).

An invitation for stroke service representatives to complete the survey was distributed electronically in September 2023 via the NHS England national stroke service newsletter, managers of regional clinical networks, and a list of neuro-interventionist contacts for each CSC. The invitation asked for completion by a person who was able to represent service views such as the local clinical lead, but did not mandate who this should be or whether there should be additional local discussion beforehand.

In order to sense-check that service responses regarding clinical scenarios were consistent with wider clinician views, the same scenarios were used in a poll of nurses and doctors from both CSC and ASC stroke services who were attending a free educational event about the latest developments in thrombectomy treatment in October 2023. The poll was conducted using the Vevox software platform (www.vevox.com) during the event and respondents did not have knowledge of the service responses.

### 3.2 Ambulance services

The ambulance survey sought views about factors which would influence when return within 24 h might be possible, tolerances for ambulance waiting times at CSC for very early patient return, and conditions when returns might and might not be deliverable. Three questions were included which were also presented to hospital services and individual clinicians about the organization of return processes, to enable comparison of all perspectives regarding the time of day when returns should happen, asking ambulances to wait at the CSC to see if patients can return immediately, and the value of auditing early return activity. The same online platform (www.castoredc.com) was used as for the hospital survey, collecting responses via multiple choice answers supplemented with free text. Questions were developed by a stroke physician (CP) and reviewed by a senior paramedic (GM) priori to dissemination.

An invitation for ambulance services to complete the survey was distributed electronically in September 2023 to members of the UK Ambulance Stroke Special Interest Group. The Group comprises representatives from all UK services who meet regularly to share information about the pre-hospital response to suspected stroke. The recipients were asked to complete the survey themselves or forward to the most appropriate person within their service.

### 3.3 Focus groups with patients and carers

The views of stroke patients and carers about early return from CSC were collected via focus groups during May 2023. Participants were volunteers recruited from regional stroke support groups in England contacted via existing community networks. Where possible, focus groups took place face-to-face at appropriate and accessible venues, and were otherwise conducted remotely using Microsoft Teams video call software. Group discussions were audio-recorded and transcribed manually. One researcher (AA) conducted the groups supported by a topic guide (Supplementary material) which was designed collaboratively by the authors to meet the project aims using their detailed knowledge of clinical care pathways and previous research experience with stroke patients and carers. The researcher initially coded data using a thematic analysis approach (Braun and Clarke, 2006). Main codes and principal themes were developed using a constant comparative method (Boeije, 2002; Tong et al., 2007). Ethical approval was granted by the Newcastle University Research Ethics Committee (30122/2022).

## 4 Results

### 4.1 Hospital stroke services

Out of a maximum of 24 CSC and 76 ASC in England there were 32 responses: 10 CSC and 22 ASC, response rates of 42 and 29%, respectively. Contributing services are listed in Supplementary material. Most responses were completed by clinical leads (*n* = 27), but also senior nurses (*n* = 3) and service managers (*n* = 2). Three CSCs reported that they had an existing patient return policy with their ASCs, but no further information was provided.

For three questions seeking views related to the processes of patient return (Table 1), the majority reported that it was acceptable for stroke patients who fully recovered after thrombectomy to be discharged directly home from the CSC without early return, whilst in situations where patients hadn't fully recovered, ASC should always accept early return of a stable patient whenever there was local capacity to do so. However, views were mixed about whether ASC staff required additional training for patients returned early, which was explained in comments from CSC as a need for understanding the management of cannulation complications post-thrombectomy and when to trigger urgent medical review in case of neurological deterioration.

**Table 1:** Hospital service survey responses describing patient return processes.

**Hospital services only (*n* = 32)**	**Yes**	**No**	**No response**
For normally independent patients who fully recover neurological function within 24 h, would it be appropriate to discharge them directly home rather than repatriate?	24 (75%)	0 (0%)	8 (25%)
Should local ASC be able to decline receiving a stable patient back if a stroke bed is available?	3 (9%)	21 (66%)	8 (25%)
Do local ASC currently require additional resources or skills to receive patients?	11 (34%)	13 (41%)	8 (25%)

### 4.2 Hospital stroke services and clinicians

At the thrombectomy education event, individual responses for the clinical scenarios were provided by 44 attendees comprising 17 stroke consultants, 10 stroke trainee doctors, seven stroke nurses, and 10 other clinicians. Their poll responses and the hospital survey responses regarding early return timescales for different clinical scenarios are summarized in Table 2.

**Table 2:** Hospital service survey and clinician poll responses describing early return suitability for clinical scenarios.

**Hospital services (*n* = 32) and clinician (*n* = 44) responses**	**Unsuitable for rapid return**<**24 h**		**Suitable for rapid return**						**No response**	
			<**4 h**		**4 to 12 h**		>**12 to 24 h**			
**Clinical scenario**	**Services**	**Clinicians**	**Services**	**Clinicians**	**Services**	**Clinicians**	**Services**	**Clinicians**	**Services**	**Clinicians**
Ischemic stroke after thrombectomy—no early improvement clinically	5 (16%)	1 (2%)	7 (22%)	17 (39%)	9 (28%)	9 (20%)	9 (28%)	16 (37%)	2 (6%)	1 (2%)
Ischemic stroke after thrombectomy—significant early improvement clinically	2 (6%)	0 (0%)	11 (34%)	32 (73%)	11 (34%)	8 (18%)	4 (13%)	4 (9%)	4 (13%)	0 (0%)
Ischemic stroke after thrombolysis alone (thrombectomy not appropriate)—no early improvement clinically	4 (13%)	1 (2%)	16 (50%)	23 (52%)	3 (9%)	3 (7%)	3 (9%)	8 (18%)	6 (19%)	9 (21%)
Ischemic stroke after thrombolysis alone (thrombectomy not appropriate)—significant early improvement clinically	2 (6%)	1 (2%)	17 (53%)	36 (82%)	4 (13%)	1 (2%)	3 (9%)	2 (5%)	6 (19%)	4 (9%)
Hemorrhagic stroke still requiring intravenous blood pressure lowering	10 (31%)	18 (41%)	8 (25%)	9 (20%)	3 (9%)	4 (9%)	5 (16%)	6 (14%)	6 (19%)	7 (16%)
Ischemic or hemorrhagic stroke unsuitable for or not requiring any acute intervention	1 (3%)	3 (7%)	19 (59%)	34 (77%)	5 (16%)	1 (2%)	1 (3%)	3 (7%)	6 (19%)	3 (7%)
Non-stroke mimic who is medically stable	4 (13%)	2 (5%)	17 (53%)	36 (82%)	1 (3%)	1 (2%)	3 (9%)	0 (0%)	7 (22%)	5 (11%)

Apart from the scenario where patients require intravenous blood pressure lowering following a hemorrhagic stroke, the majority of services and clinicians supported the return of patients within 24 h of CSC admission. Respondents who did not support early return of hemorrhagic stroke cases commented that there was a risk of losing blood pressure control during transfer, and being at high risk of deterioration, they might still require urgent neurosurgical review at the CSC.

After thrombectomy, services tended to prefer patient returns to occur later than clinicians during the first 24 h but both groups were more cautious when thrombectomy did not result in clinical improvement. Respondents commented that these patients may still be at risk of cerebral oedema and require neurosurgical intervention at the CSC such as hemicraniectomy, and that some patients may have issues associated with thrombectomy such as bleeding from a cannulation site or anesthetic complications. After thrombolysis alone, a larger proportion of all respondents were in favor of return < 4 h with or without improvement, although some were still concerned about monitoring for hemicraniectomy. Responses were similar for patients who were not suitable for any emergency intervention and non-stroke mimic scenarios, with the majority being in support of return < 4 h. Some service respondents were concerned about the difficulty of obtaining a neurological review for non-stroke mimic patients returned to a local hospital if the diagnosis was not already obvious, but others recognized that these patients are already regularly admitted to local hospitals and ASC, and local processes should already be in place for liaison with other specialties as needed.

### 4.3 Ambulance services

Eleven ambulance services completed surveys (see Supplementary material), a response rate of 79% across the UK. Two responses were from UK devolved nations (i.e., the Scottish and Welsh Ambulance Services), but the data were still included because these services are part of the UK ambulance network and patient return issues are still very relevant within their similar healthcare systems. Surveys were completed by nine senior paramedics (three with a specific remit for stroke care) and two medical directors. Only one respondent was aware of a formal policy for patient return from CSCs within their service boundary.

For three questions seeking views on ambulance-specific organizational aspects of early patient return (Table 3), the majority supported a specific payment for this activity, illustrated by comments reflecting that it is not usually part of service workload and might be particularly disruptive for the ability to respond to new emergency calls if vehicles were performing patient returns over longer distances. There was support for subcontracted ambulance providers assisting with early returns, but uncertainty whether a hospital escort would be needed.

**Table 3:** Ambulance service survey responses describing the organization of patient returns.

**Ambulance services only (*n* = 11)**	**Yes**	**No / not sure**
Should rapid patient return from regional to local stroke units attract a specific payment?	7 (64%)	4 (36%)
Is there a role for private / sub-contracted ambulance providers to assist with early repatriation of patients from regional stroke centers?	8 (73%)	**3 (27%)**
Should a hospital escort accompany any specific patient groups?	6 (54%)	5 (46%)

When asked about influences upon the possibility of early patient return being accommodated at the time when it is requested (Table 4), the majority reported that considerations would include the travel time between central and local hospital sites (73%), immediate overall pressures on the ambulance service (91%), the time of day when a request is made (73%) and the type of crew (qualified paramedics or technicians) that would be available to make the transfer (73%). However, the day of the week was not considered to be as important (36%), and there was no particular advantage if an ambulance was already heading to the CSC with another patient from the relevant local hospital area (27%). Free text comments for these two items explained that the ambulance service is required to provide the same emergency response every day, and that even if a crew at a CSC is free for another job which coincides with a local patient being available for early return, it cannot be guaranteed that this would be a priority for that resource.

**Table 4:** Ambulance service survey responses describing influences upon patient returns.

**What ambulance factors might be relevant for enabling patient return within 24 h of regional center admission? (*n* = 11)**	**Likely to be relevant**	**Unlikely to be relevant**
The travel time between central and local hospital sites	8 (73%)	3 (27%)
Immediate overall pressures on the ambulance service	10 (91%)	1 (9%)
The time of day when a request is made	8 (73%)	3 (27%)
The type of crew that is available to make the transfer	8 (73%)	3 (27%)
The day of the week when a request is made	4 (36%)	7 (64%)
If an ambulance is already heading to the center with another patient from the relevant local hospital area	3 (27%)	8 (73%)

### 4.4 Hospital stroke services, clinicians, and ambulance services

Regarding times when an early return process should be provided (Table 5), hospital clinicians (66%) and ambulance services (46%) tended to favor 24 h, but hospital services preferred between 8 am and 8 pm only (41%). Their view reflected concerns that patients might experience complications of thrombectomy or cerebral oedema overnight at ASC when less medical cover is available, and the greater challenge of creating local bed capacity during the night.

**Table 5:** Hospital service, clinician and ambulance service preferences for the timing of patient returns.

**What times of day should return of patients be considered?**	**24 h**	**8 am−4 pm only**	**8 am−8 pm only**	**8 am–midnight only**	**No response**
Hospital stroke services (*n* = 32)	6 (19%)	3 (9%)	13 (41%)	2 (6%)	8 (25%)
Hospital clinicians (*n* = 44)	29 (66%)	2 (4%)	3 (7%)	2 (4%)	8 (18%)
Ambulance services (*n* = 11)	5 (46%)	0 (0%)	3 (27%)	0 (0%)	3 (27%)

Whilst hospital services (78%) and clinicians (73%) supported immediate return in the same ambulance for patients who significantly improved during initial transfer from a local hospital to a CSC (Table 6), the ambulance services were not in favor (27%) and commented that they were not confident that hospital assessment could make this decision within 30–60 min, and would only consider this option if there was evidence that a 30 min turn around was possible. However, all three groups agreed that audit data should be collected that describes how services return stroke patients from CSC.

**Table 6:** Hospital service, clinician, and ambulance service responses describing patient return processes.

	**Hospital services (*****n*** = **32)**			**Hospital clinicians (*****n*** = **44)**			**Ambulance services (*****n*** = **11)**		
	**Yes**	**No**	**No response**	**Yes**	**No**	**No response**	**Yes**	**No**	**No response**
For patients who significantly improve during initial transfer from a local hospital to a CSC, and they do not require any emergency treatment at the CSC, would it be acceptable to perform an assessment within 30–60 min and return them in the same ambulance?	25 (78%)	0 (0%)	7 (22%)	32 (73%)	0 (0%)	12 (27%)	3 (27%)	8 (73%)	0 (0%)
Should audit data be collected that describes how services return stroke patients?	21 (66%)	2 (6%)	9 (28%)	35 (80%)	2 (4%)	7 (16%)	8 (73%)	3 (27%)	0 (0%)

### 4.5 Focus groups with patients and carers

Thirty-three participants participated: 29 patients (20 male and nine female) and four carers (one male and three female). There were three focus groups in total: two were face to face with participants in North East (*n* = 15) and North West England (*n* = 12), and a further online group with participants from both regions (*n* = 5). One individual interview took place with a patient who could not attend a focus group. Recordings lasted between 56 and 121 min.

Three principal themes were identified: “support for early return;” “suitable circumstances for early return” and the “importance of communication.”

#### 4.5.1 Support for early return

Many participants saw early return as a rational solution to give all appropriate patients the opportunity to access thrombectomy, and that it was also beneficial to ensure that patients are closer to their families as soon as possible. Several participants felt that early return was “not ideal” and might be unsettling for patients, however they understood it might be the only solution for maintain thrombectomy emergency beds and other patients may feel differently. A few participants expressed a concern that early return may be carried out in the interests of meeting hospital targets, rather than to do what is best for individual patients.

#### 4.5.2 Suitable circumstances for early return

Participants proposed that early return was acceptable in general if (1) it did not pose any risk to long term outcomes (2) the same quality of care was available at a local unit (including not just going back into a local ED queue) and (3) that return decisions are made on a case-by-case basis sensitive to each individual patient's circumstances including those listed below.

##### 4.5.2.1 Clinical status

Participants were unanimous that no patient should be moved unless they were clinically stable and it was unlikely that the return ambulance journey would exacerbate symptoms.

##### 4.5.2.2 Age

Some participants were concerned that multiple transfers in a short space of time may be disorientating for older patients. Other participants felt that early transfer was acceptable for all patients provided they had received the appropriate assessment, treatment, and care at the regional thrombectomy center before being transferred.

##### 4.5.2.3 Journeys

Several participants suggested that early return would be more acceptable/appropriate for patients who hadn't already had multiple long ambulance journeys as part of their care; for example, patients previously brought by ambulance from a very rural location to an ASC before being transferred to a CSC.

##### 4.5.2.4 Patient preferences

Some participants felt that patients and their families should have a choice in whether they are taken back to a local unit soon after assessment, treatment, and care at a thrombectomy center. Other participants expressed they would prefer for doctors to make an appropriate decision on their behalf, with some stating that patients should not be given the opportunity to make a decision on something they potentially do not fully understand.

##### 4.5.2.5 Wider patient circumstances

Several participants highlighted that early return decisions should be sensitive to wider patient circumstances. The examples highlighted by participants included patients with mental health issues and those without an external support network.

#### 4.5.3 Importance of communication

Participants highlighted that the experience of early return would be dependent upon efficient communication between all healthcare providers, patients and their families. Participants expressed that if early return was explained to them in a sensitive manner by the care team and they understood why it was happening, that it was not a risk to their recovery and that it was in the interests of best overall care, they would be amenable. No participants were concerned about the prospect of night-time transfers as long as this was communicated in advance, but some participants were concerned about delays which could occur at any time after this communication due to inter-hospital “bed politics.”

## 5 Discussion

This mixed methods evaluation sought service, clinician and public views regarding relevant aspects of early return processes for patients displaced at CSC as a result of redirection pathways intended to optimize emergency treatment and improve outcomes. There was overall support for the concept of early return to improve patient flow through thrombectomy services and reduce displacement from local care, but this was not universal for all situations and the results suggest several important organizational, clinical and patient-focused considerations for implementation. Recommended actions for services and networks are listed in Box 1.

Box 1 **Organizational, clinical, and patient-focused recommended actions for implementation of early patient return from CSC.**

**Organizational actions**

- Seek regional network agreement about the timing and travel boundaries for early returns.
- Confirm ongoing local care arrangements for all returned patients including mimic cases.
- Address training needs of ASC staff including post-thrombectomy management.
- Develop early return audit standards and include return data in performance reports.

**Clinical actions**

- Agree and formalize return timelines and shared care protocols for the main clinical scenarios of thrombectomy, thrombolysis only, hemorrhagic stroke and non-stroke mimics.
- Clarify when an escort is needed and actions to take should returning patients deteriorate.
- Standardize communication between CSC, ASC and ambulance services to co-ordinate safe and efficient return of patients.

**Patient-focused actions**

- Ensure public representation in planning and evaluation of early return pathways.
- Personalize early return decisions for individual patients including consideration of clinical status, symptoms and journey length.
- Standardize communication with patients and families about early return processes, and pro-actively seek views on treatment escalation.
- Seek patient feedback about early return experiences.

There was support for the return of patients within 24 h of thrombectomy, but the preferred timing was >4 h, especially if patients had not yet experienced clinical improvement. Service respondents were more cautious than clinicians, possibly reflecting a greater awareness of organizational governance responsibilities. Although there is a good volume of published data describing complications after thrombectomy, there are only limited descriptions of the implications for early return pathways. The most relevant and comprehensive report is by Griffin et al. (2020), which describes the experience of transferring 352 patients locally (< 90 min drive; median distance 43 km) and 83 remotely (>90 min drive; median distance 217 km) across Ireland between January 2016 to June 2018 after anterior circulation thrombectomy. It is important to note that most procedures were under local anesthetic +/- conscious sedation, and only eight received a general anesthetic. In the local group, 322/352 (91%) were returned immediately post-procedure, whilst in the more remote group, 56/83 (67%) were repatriated within 24 h. Longer CSC admission was main being due to CSC clinician preference for longer observation, intracranial hemorrhage, decompressive hemicraniectomy, and/or the need for ICU admission. An overall total of 10 returned patients (2%) required re-admission to the CSC due to issues related to their stroke or thrombectomy. Although this report is reassuring, the data will reflect the population and care received, and may not be directly applicable to other settings. For instance, national audit data for England and Wales from April 2022 to March 2023 (Sentinel Stroke National Audit Programme, 2024) show that 75% of procedures involved a general anesthetic, whilst ~10% were admitted to ICU/HDU afterwards, which may impact upon the volume suitable for very early return.

When planning return pathways, it is important to recognize that complication rates after thrombectomy depend upon the setting and definitions applied, as illustrated by a previous literature review which found variation between 4 and 29% in trials and 7–31% in prospective studies/registries (Balami et al., 2018). However, most adverse events are likely to be immediately obvious and much less likely to occur after the early return of stable patients. It is possible that the future risk of complications will be changed by ongoing expansion of thrombectomy-eligible populations to include patients with later presentations (Nguyen et al., 2022), greater baseline radiological changes (Sarraj et al., 2024) and less severe symptoms (McCarthy et al., 2021), as well as changes in devices and anesthetic processes. Therefore, ongoing collection of post-thrombectomy care data is needed, with local evaluation to ensure that return pathways remain appropriate for the population served.

The majority support for return < 4 h after thrombolysis may reflect greater confidence that local teams can manage the consequences since they already provide this therapy, whereas thrombectomy is only available at CSC. Although ASC also routinely initiate intravenous blood pressure lowering for hemorrhagic stroke, the greater caution for this scenario may illustrate a higher risk of deterioration and the practical challenge of returning patients whilst receiving a titrated dose intravenous infusion. It was however generally agreed that patients with a stroke unsuitable for any immediate intervention or with a stable non-stroke mimic condition could be considered for return < 4 h.

The clinical scenarios used to collect views about early return timescales reflected common combinations of diagnosis, treatment and outcome information but further details were omitted in order to improve survey and poll response rates. In reality, it is expected that return decisions for individual patients would reflect more nuanced consideration of their health status, investigation findings, procedures performed and immediate prognosis. For instance, patients who are found to have a vascular malformation as a cause of a hemorrhagic stroke may requiring ongoing treatment at a CSC, whilst those very severely affected by stroke and requiring palliation might be considered “too unwell” to be transferred. The importance of this personalization of care was expressed in a number of ways during the focus groups, where patients and carers were concerned about protocol-based bed management processes and targets taking precedence over clinical care and personal comfort. To provide reassurances that patient experience would not be overlooked during the planning of early return pathways, clinical networks should include public representation and seek patient and carer feedback following implementation, particularly in more geographically dispersed regions.

From a patient flow perspective, even if CSC assessment could be performed very quickly, ambulance services still did not support a “front door return” approach because of the potential wider impact on their patient handover and emergency response targets. Another point of disagreement was whether returns should occur overnight, which was supported by ambulance respondents and public participants but not hospitals. It may be helpful to undertake additional exploration about barriers to overnight movement of patients, as this could usefully facilitate thrombectomy capacity for the following morning, especially for wake-up stroke presentations. An economic evaluation of early return pathways including front door and overnight options would also be valuable, so that policies and targets can reflect approaches which are best for the wider care system.

There were mixed survey responses about whether ASC staff require additional training to meet the needs of early return patients but the focus groups revealed strong views that nobody should be moved to a setting which was unable to provide appropriate care. Before implementation of early return, services should undertake assessment of the local knowledge and skills needed to safely host return pathways, with clear delegation of clinical responsibilities. The return or repatriation of patients from regional centers to local teams for ongoing care is not unique to stroke and lessons could be learnt from other specialties. Due to the challenging combination of people, settings, and tasks, previous authors examining mixed inter-hospital transfers have suggested applying human factor frameworks to plan safe pathways such as the Systems Engineering Initiative for Patient Safety (SEIPS) model to recognize the interaction between patients and clinicians, tasks, technology, physical environment, and socio-organizational conditions (Yu et al., 2024). Future research should use this type of framework to assist with the development and monitoring of early return pathways, and ensure that there is engagement with all members of the multidisciplinary clinical team (e.g., nurses) across ASC as well as CSC.

Ambulance services confirmed that the ability to provide early returns would depend upon a number of dynamic influences such as time of day and travel distance, and in order to increase capacity to prioritize this function there was support for a specific early return payment. The matching of emergency response supply to demand is widely recognized as a challenge (Al-Azzani et al., 2021), and clinical networks should consider whether it is possible to predict the need for ambulance return journeys within the next 24 h. If linked data are routinely collected about CSC admissions and returns, it would be possible to undertake a future modeling exercise to understand the demand for early returns and the relative impact of different influences so that efficient and economically viable pathways can be developed.

It is important to acknowledge the limitations of this preliminary work, including the relatively low number of hospital services that completed surveys and the possible influence of responder bias upon the results. For example, since the clinicians polled were voluntarily attending an educational event, they might represent a more pro-active group and their views might be more in favor of early patient return processes than services in general (as illustrated by the greater proportion of clinician responses supporting return < 4 h for some clinical scenarios). There were participants who did not respond to some questions in the surveys and poll. Whilst this can reduce confidence in the results, a lack of response may also indicate that the questions posed scenarios and processes that had not been considered previously or for which there was not a definitive view, thereby reinforcing the importance of undertaking further research on this topic. As an initial exploration of this topic we restricted the proposed scenarios to a small number of commoner possibilities, such as patients who received or did not receive thrombectomy, with or without complications. Future work should seek views from a larger number of services and clinicians, with specific exploration of clinical scenarios where there was some disagreement on return timescales, with more detailed exploration of patient subgroups. We did not separately present survey responses from CSC and ASC, or attempt to report an overall regional service network view, as this would have increased the complexity of reporting and reduced the number of service-type responses for each answer, but it is possible that there are different views between these two types of centers which should be understood in order to achieve effective implementation of early return processes. The focus groups were undertaken as an initial exploration of patient and carer views, but the geographical limitations of this workstream should be noted in case this has a bearing upon previous care experiences or concerns about early return processes, and it would be valuable to also seek views from patients and carers who have had recent experience of these pathways.

## 6 Conclusion

In conclusion, the early return of selected patients from CSC to ASC is seen by professionals, patients and carers as an acceptable approach to improving the flow of patients through centralized thrombectomy services. There was general agreement amongst contributors that return decisions mainly reflect individual clinical status, workforce skills, co-operation between centers and the overall system capacity to offer safe specialist care. However, there were some mixed views both within and between stakeholder groups about the timing and process of achieving returns, which highlights the importance of seeking agreement across clinical networks and the national sharing of relevant metrics for ongoing improvement of emergency stroke care pathways.

## Data Availability

The datasets presented in this article are not readily available because, data from the hospital survey, clinician poll, and ambulance survey are presented in the article. Ethics approval did not include sharing the focus group data outside of the project due to risks of participant identification. Requests to access the datasets should be directed to: strokeresearch@newcastle.ac.uk.
