# Supplementary material for: Professional and public views about early return of patients from Comprehensive Stroke Centers to local Acute Stroke Centers in England following displacement by emergency care pathways
Source: Front Stroke. 2024 Nov 20;3:1431799. doi: 10.3389/fstro.2024.1431799 (PMC12802610; doi:10.3389/fstro.2024.1431799)
Supplement: Supplementary file 1 [file Data_Sheet_1.docx]

**SUPPLEMENTARY MATERIAL**

**1) Public interview topic guide**

*Introduction*

- Introductions
- Talk through information sheet and consent form
- Explain recording of interview/ focus group
- Any questions or concerns before proceeding
- Obtain written consent or confirm plan for verbal consent

*Presentation*

- Brief PowerPoint presentation delivered by researcher detailing the background and context

*Scenarios, questions and discussion*

- Participant thoughts on benefits/ drawbacks of early patient return
- Present scenarios with associated prompts and questions

1. Imagine you are a patient who has just received thrombectomy having travelled on the ‘drip-ship’ pathway. How would you feel about being transferred back to your local hospital rapidly following the procedure? Consider that this would mean a third ambulance journey for you in a short space of time (12-24h)

- How would you feel about receiving care at a hospital closer to home compared to remaining at the specialist centre? *Consider that staying at the thrombectomy centre may mean reduced capacity at the centre for other stroke patients.*
- Would your thoughts and feelings still apply if the patient was a friend/loved one/family member?
- What timescale for going back to the local hospital ‘early’ feels acceptable? Would you be happy to accept immediately following the thrombectomy, within 6-12 hours or 12-24 hours?
- If you feel early return is acceptable, would this apply at any time of the day or night? What about if the patient was a friend/loved one/ family member?
- What other factors might influence your view?

1. Consider that you are a patient who has been transferred to a thrombectomy centre via the ‘drip and ship’ pathway. You arrive at the centre and it is discovered that you do not need thrombectomy as the ‘clot busting’ drugs have taken effect.

- How would you feel about being rapidly transferred back to your local hospital for on-going treatment? *Consider that staying at the thrombectomy centre may mean reduced capacity at the centre for other patients in need of thrombectomy treatment. Consider also that this would be your third ambulance journey in a short amount of time (12-24h)*
- Would your thoughts and feelings still apply if the patient was a friend/ loved one/ family member?
- What timescale for going back to your local hospital ‘early’ feels acceptable? Would you be happy to accept immediately after its decided you do not need thrombectomy, within 6-12 hours or 12-24 hours?
- If you feel early return is acceptable, would this apply at any time of the day or night? What about if the patient was a friend/loved one/ family member?
- What other factors might influence your view?

1. Imagine you are a patient who has just received thrombectomy having travelled on an ‘ambulance redirection’ pathway. How would you feel about being transferred to your local hospital early following the procedure?

- How would you feel about receiving care at a hospital closer to home compared to remaining at the specialist centre? *Consider that staying at the thrombectomy centre may mean reduced capacity at the centre for other patients in need of thrombectomy treatment.*
- Would your thoughts and feelings still apply if the patient was a friend/loved one/family member?
- What timescale for going to your local hospital ‘early’ feels acceptable? Would you be happy to accept immediately following the thrombectomy, within 6-12 hours or 12-24 hours?
- If you feel early return is acceptable, would this still apply at any time of the day or night? What about if the patient was a friend/loved one/ family member?
- What other factors might influence your view?

1. Consider that you are a patient who has been redirected to a thrombectomy centre on an ambulance redirection pathway. You arrive at the centre and it is discovered that you do not need thrombectomy. This may be because it’s not the right type of stroke for this treatment or the problem is not actually a stroke.

- How would you feel about being rapidly transferred to your local hospital for on-going treatment? *Consider that staying at the thrombectomy centre may mean reduced capacity at the centre for other patients in need of thrombectomy treatment.*
- Would it make any difference whether your diagnosis was stroke or another condition which was initially thought to be a stroke?
- Would your thoughts and feelings still apply if the patient was a friend/ loved one/ family member?
- What timescale for being transferred to your local hospital ‘early’ feels acceptable? Would you be happy to accept immediately after initial assessment and/or treatment, within 6-12 hours or 12-24 hours?
- If you feel early return is acceptable, would this apply at any time of the day or night? What about if the patient was a friend/loved one/ family member?
- What other factors might influence your view?

1. Reflect on the views you have shared so far and consider if they would be any different if your local hospital was at a considerable distance from a thrombectomy centre (1hr +). With this in mind:

- Would a long journey time change your views about an early return?

- Would a long journey time change your view about the timescale for returning?

- Does any distance feel unacceptable for early returns?

- Would it make any difference whether it was daytime or night?

*Close interview/ focus group* – Thank participants, next steps

**2) Hospital stroke services that responded to the online survey**

a) Services stating that they provide thrombectomy

- Aintree hospital, LUHFT
- Hull Teaching Hospitals
- Lancashire Teaching Hospitals NHS Trust
- North Bristol Trust
- Nottingham University Hospital
- Queen Elizabeth Birmingham
- Royal Sussex County Hospital, University Hospitals Sussex NHS Foundation Trust
- Salford Royal Hospital, Northern Care Alliance NHS Foundation Trust
- University Hospital of Coventry & Warwickshire NHS Trust
- University Hospital Southampton

b) Services stating that they do not provide thrombectomy

- Chesterfield Royal Hospital
- Doncaster and Bassetlaw Teaching Hospitals
- East Sussex Hospitals Trust
- Frimley NHS Foundation Trust, Frimley Park Hospital
- Great Western Hospital
- James Paget NHS Foundation Trust
- Mid and South Essex Hospital Trust- Broomfield
- Mid Yorkshire Hospitals NHS Trust
- MSE Basildon, Southend and Broomfield
- Northumbria Healthcare NHS Foundation Trust
- Northwick Park Hospital. London north west hospital university NHS
- Queen Elizabeth Hospital, Norfolk
- Royal Devon NHS Trust
- Royal Hampshire County Hospital, Hampshire Hospital Foundation Trust
- Royal Wolverhampton NHS Trust
- St Richard's Hospital University Hospital Sussex
- Sunderland and South Tyneside FT
- University Hospital North Durham, County Durham and Darlington NHS Foundation Trust
- University Hospital of North Tees
- University Hospitals Dorset
- West Suffolk NHS Foundation Trust
- Worthing Hospital

**3) Ambulance services that responded to the online survey**

- East Midlands Ambulance Service
- East of England Ambulance Service
- North East Ambulance Service
- North West Ambulance Service
- Scottish Ambulance Service
- South Central Ambulance Service
- South East Coast Ambulance
- South Western Ambulance Service
- Welsh Ambulance Service NHS Trust
- West Midlands Ambulance Service
- Yorkshire Ambulance Service
